# Influence of Engine‐Driven NiTi Files on the Effectiveness and Technical Quality of Endodontic Treatment Performed by Undergraduate Students: A Systematic Review and Meta‐Analysis

**DOI:** 10.1111/iej.70056

**Published:** 2025-10-26

**Authors:** Monique Corrêa Rocha Ferrari Barbosa, Elidiane Elias Ribeiro, Karem Paula Pinto, Ana Flávia Almeida Barbosa, Emmanuel João Nogueira Leal da Silva, Luciana Moura Sassone

**Affiliations:** ^1^ PROCLIN Department, School of Dentistry Universidade do Estado do Rio de Janeiro (UERJ) Rio de Janeiro Brazil; ^2^ Department of Endodontics, School of Dentistry Grande Rio University (UNIGRANRIO) Rio de Janeiro Brazil

**Keywords:** dental education, education, endodontic, engine‐driven instrumentation, root canal therapy, students

## Abstract

**Background:**

In recent years, many universities have incorporated engine‐driven nickel‐titanium (NiTi) instruments into their undergraduate endodontic programs, aiming to enhance the efficiency and precision of root canal treatments. While these techniques might offer advantages, concerns remain regarding their implementation, particularly in ensuring adequate training and supervision to prevent potential procedural errors that could compromise treatment outcomes.

**Objectives:**

This systematic review and meta‐analysis aimed to evaluate the quality of clinical root canal treatments performed by undergraduate students, comparing outcomes between the use of engine‐driven NiTi files and stainless‐steel hand files.

**Methods:**

The search was conducted across four electronic databases up to April 2025. The selection process adhered to the Population, Intervention, Comparison, Outcomes and Study (PICOS) criteria, including clinical studies evaluating the quality of root canal treatments performed by undergraduate students using engine‐driven NiTi files versus stainless‐steel hand files. Study quality was assessed using the Risk of Bias 2 (RoB‐2) tool for randomised clinical studies and Risk of Bias in Non‐randomised Studies of Interventions (ROBINS‐I) for nonrandomized studies. Meta‐analyses were performed to compare the prevalence of adequate root canal treatment quality, root canal filling quality, and procedural errors between the two techniques, using RevMan 5.3 software. The Grading of Recommendations Assessment, Development, and Evaluation (GRADE) tool was applied to determine the overall certainty of evidence.

**Results:**

Ten studies were included, with most demonstrating a high risk of bias. Meta‐analyses revealed that engine‐driven NiTi root canal preparation with both rotary and reciprocating instruments resulted in significantly higher quality of root canal treatment, improving root canal filling quality, and reducing the incidence of ledge formation compared to stainless‐steel hand files. However, no significant differences were found between the techniques regarding overfilling, perforations, apical transportation, or instrument separation. The certainty of evidence was rated as low.

**Conclusions:**

Engine‐driven NiTi instruments improve the quality of root canal treatments performed by undergraduate students, particularly by enhancing filling quality and reducing ledge formation without increasing other iatrogenic risks. Nevertheless, these conclusions are based on evidence of low certainty.

**Trial Registration:**

This systematic review was registered in the PROSPERO database (CRD42024600660)

## 1 Introduction

Endodontic education has undergone significant evolution to meet contemporary challenges; however, many institutions still rely on traditional teaching methods that may not fully incorporate recent advancements in the field (Nagendrababu et al. 2024). Current educational guidelines emphasize the importance of preparing students to accurately diagnose pulpal and periradicular conditions while providing adequate clinical experience to ensure competence in delivering effective root canal treatments (Baaij et al. 2024; Sadr et al. 2021). Despite these efforts, studies highlight that dental students often perceive endodontics as one of the most challenging disciplines in dentistry, largely due to its technical demands and steep learning curve (Friedlander et al. 2023).

Recent developments in endodontic instruments have significantly influenced teaching practices. Advances in endodontic science, including a deeper understanding of root canal system anatomy and innovations in material technology, have positioned Nickel‐Titanium (NiTi) instruments as a cornerstone of modern endodontics. These instruments, with their superior flexibility enhanced by advanced metallurgical treatments, allow for safer and more efficient preparation of curved and complex canals compared to traditional stainless‐steel files (Garip and Günday 2001; Chaniotis and Ordinola‐Zapata 2022). Consequently, these technological improvements have greatly enhanced the technical quality of root canal procedures while minimizing procedural errors, such as canal transportation and ledge formation, which are common among less experienced practitioners (Garip and Günday 2001; Peralta‐Mamani et al. 2019). Furthermore, a recent meta‐analysis demonstrated the superior effectiveness of engine‐driven NiTi instruments over conventional stainless‐steel files in achieving successful outcomes, particularly in cases of apical periodontitis followed for at least 1 year (Buerklein and Arias 2023). These findings not only highlight the clinical benefits of adopting modern endodontic technologies but also align with the evolving educational standards aimed at equipping students with the skills required for contemporary practice. As a result, the integration of engine‐driven instrumentation into dental school curricula has become increasingly widespread, reflecting its transformative impact on root canal treatment and endodontic education (Almanei 2018; Nagendrababu et al. 2024).

While the benefits of engine‐driven NiTi instruments in root canal treatment are well documented (Chaniotis and Ordinola‐Zapata 2022), and their use has become routine among general practitioners and specialists (Cheung and Parashos 2023; Cheung et al. 2023; Logsdon et al. 2020; Madarati and Habib 2018), incorporating them into undergraduate education presents unique challenges. One of the primary barriers is the significant financial investment required for these instruments, which can be particularly prohibitive for institutions operating in resource‐limited settings or in countries with constrained financial infrastructures (Hänni et al. 2003). Additionally, the lack of experience can increase the risk of complications, such as instrument separation, over‐instrumentation, or canal transportation, all of which could compromise the quality of treatment and patient outcomes. These challenges underscore the critical need for comprehensive and carefully structured training programs that equip students with the necessary skills, confidence, and theoretical understanding to safely and effectively utilise these instruments (Spångberg 2001).

Despite these obstacles, the potential benefits of engine‐driven NiTi instruments in undergraduate endodontic education cannot be overlooked. If evidence suggests that their use significantly improves treatment outcomes even at the undergraduate level, this could represent a transformative change in dental education, encouraging even resource‐constrained institutions to reconsider their investment priorities. The long‐term advantages of better‐prepared graduates, capable of delivering higher‐quality care, may outweigh the initial financial and logistical hurdles. However, to make informed decisions, it is essential to critically evaluate the existing data on their clinical efficacy when used by students. Therefore, this systematic review and meta‐analysis aims to assess the impact of engine‐driven NiTi instruments on the quality of root canal treatment performed by undergraduate students.

## 2 Methods

### 2.1 Protocol and Registration

The protocol for this systematic review was registered with the PROSPERO database (registration number CRD42024600660) and adhered to the Preferred Reporting Items for Systematic Review and Meta‐Analysis (PRISMA) guidelines (http://www.prisma‐statement.org).

### 2.2 Focused Question

This systematic review was guided by the following focused question: *Does the use of engine‐driven NiTi instruments improve the quality of root canal treatment performed by undergraduate students compared to stainless‐steel hand instrumentation?* The null hypothesis tested was that there would be no difference in the quality of root canal treatment performed by undergraduate students using engine‐driven or stainless‐steel hand instrumentation.

### 2.3 Search Strategy

The systematic search was performed by two independent reviewers (M.C.R.F.B and E.E.R.) across four electronic databases: PubMed, Scopus, Web of Science, and Cochrane. Additionally, grey literature was searched using Google Scholar. The search was conducted on April 13, 2025, without language restrictions or filters. The selection of descriptors was informed by the most frequently cited terms in previous publications on this topic. Boolean operators ‘AND’ and ‘OR’ were used to refine the search strategy, following the syntax rules specific to each database (Table S1).

### 2.4 Study Selection

#### 2.4.1 Inclusion Criteria

The inclusion criteria for this systematic review were established using the PICOS strategy, as follows:

P (population): Patients submitted to root canal treatment performed by undergraduate students.

I (intervention): Engine‐driven NiTi primary non‐surgical root canal treatment (rotary or reciprocating).

C (comparison): Stainless‐steel hand root canal preparation.

O (outcome): Quality of root canal treatment (evaluated by clinical and radiographic assessments, including postoperative pain, periapical healing, the length and homogeneity of root canal fillings, and the prevalence of procedural errors).

S (study design): Randomised and non‐randomised clinical studies.

This systematic review evaluated clinical studies comparing the performance of stainless‐steel hand files and engine‐driven NiTi instruments used by undergraduate dental students. The comparison was based on clinical and radiographic assessments. Clinical evaluations focused on signs and symptoms, including postoperative pain, while radiographic assessments examined factors such as the quality of instrumentation and root canal filling, periapical healing, and the occurrence of procedural errors, including perforations, instrument separation, ledge formation, and apical transportation.

#### 2.4.2 Exclusion Criteria

Non‐clinical studies were excluded. Clinical studies that did not compare stainless‐steel hand instruments with engine‐driven NiTi instruments were excluded, as well as studies where the intervention was performed by specialists, residents, or dentists. Additionally, articles not aligned with the study's theme, such as in vitro studies, ex vivo studies, questionnaire‐based studies, animal studies, case reports, case series, letters to the editor, opinions, and reviews, were also excluded.

The articles retrieved from the initial search were imported into EndNote X9 software (Thomson Reuters) for duplicate removal. The remaining articles were then exported to Rayyan (https://www.rayyan.ai) for systematic screening. Titles and abstracts were independently analysed by two reviewers (M.C.R.F.B. and E.E.R.), and potentially relevant studies were thoroughly assessed for eligibility. In cases of discrepancy, a third co‐author (K.P.P.) was consulted to make the final decision. A previous calibration of the reviewers involved a pilot screening phase in which the two reviewers independently assessed a sample of 50 studies using the predefined inclusion and exclusion criteria. Discrepancies were discussed and resolved to ensure a consistent interpretation of the criteria.

### 2.5 Data Extraction

Two authors (M.C.R.F.B and E.E.R.) independently extracted data from the included studies. The collected information included the author, year and country of publication, the stainless‐steel hand file and sample size, the engine‐driven NiTi file and sample size, the measured outcomes and results, and the main findings. Additionally, patient demographics (age), the cause of root canal treatment, tooth type, case complexity, pulpal diagnosis, hand instrumentation technique, irrigant solution, obturation technique, root canal filling material, restorative material used and the follow‐up time were also recorded. The authors of the included studies were contacted by e‐mail on three occasions when the crude data was not available.

### 2.6 Quality Assessment

The quality of the included studies was assessed by two independent authors (M.C.R.F.B and E.E.R.) using the RoB‐2 for randomised studies (Sterne et al. 2019) and ROBINS‐I for nonrandomized studies (Sterne et al. 2016). The RoB‐2 tool evaluates the following domains: randomization process, deviations from intended interventions, missing outcome data, measurement of outcomes and selection of reported results. The ROBINS‐I tool evaluates the following domains: confounding, selection of participants, classification of interventions, deviations from intended interventions, missing data, measurement of outcomes, and selection of the reported results. Each domain was categorised as having ‘low risk’, ‘some concerns’, or ’high risk’ of bias. The overall risk of bias for a study was classified as ‘low risk’ if all domains were assessed as ‘low risk’, ‘moderate risk’ if one or more domains were rated as ‘some concerns’, and ‘high risk’ if any domain was classified as ‘high risk’.

### 2.7 Meta‐Analysis

The meta‐analyses were performed using RevMan software (Version 5.3, The Cochrane Collaboration) and forest plots were conducted to compare the prevalence of overall adequate quality of root canal treatment, adequate quality of root canal filling, and procedural errors, between engine‐driven and stainless‐steel hand instrumentation.

The prevalence was recorded as dichotomous data. The estimate of the intervention's effect was expressed as risk ratios (RR) along with 95% confidence intervals (CIs). The statistical heterogeneity among the studies was evaluated using the *I* ^2^ index, and in cases of low heterogeneity (*I* ^2^ ≤ 50%), the fixed‐effect model was employed, while random‐effect models were employed in cases of moderate or high heterogeneity. Publication bias was assessed both visually, using funnel plots, and quantitatively, through Egger's regression test, with statistical significance set at *p* ≤ 0.05, provided there were at least 10 studies included in the meta‐analysis.

### 2.8 Grading of Evidence

The level of evidence was evaluated using the Grading of Recommendations, Assessment, Development and Evaluation methodology through GRADEpro Guideline Development Tool (McMaster University; Guyatt, Oxman, Akl, Kunz, et al. 2011). This tool has 5 domains: Risk of bias, Inconsistency, Indirectness, Imprecision and Publication bias. The classification of each domain was determined as ‘not serious’, ‘serious’ or ‘very serious’, and their overall certainty of evidence was graded into one of four levels: very low, low, moderate or high.

In assessing the ‘Risk of Bias’ domain, the following factors were considered: patient selection criteria, measurement of the intervention and outcomes, control of confounding factors in study design or statistical analysis, and adequacy of follow‐up (Guyatt, Oxman, Vist, Kunz, et al. 2011). Ratings were categorised as ‘not serious’ if more than three criteria received a ‘no’ response in all studies, ‘serious’ if two to three ‘no’ responses were observed, and ‘very serious’ if one to two ‘no’ responses were noted.

For the ‘Inconsistency’ domain, the consistency of study effects was evaluated by reviewing point estimates, confidence intervals, and heterogeneity (Guyatt, Oxman, Kunz, Woodcock, et al. 2011b). A ‘not serious’ rating was assigned if all studies showed consistent results, ‘serious’ if some studies had inconsistent findings, and ‘very serious’ if most studies exhibited inconsistent outcomes.

In the ‘Indirectness’ domain, differences in the target population, the nature of the intervention, and the relevance of reported outcomes to the patient population were considered (Guyatt et al. 2011a). A ‘not serious’ rating was given if all studies had more than 3 ‘no’ responses for these criteria, ‘serious’ if there were 2–3 ‘no’ responses, and ‘very serious’ if 1–2 ‘no’ responses were noted.

For the ‘Imprecision’ domain, factors such as sample size and the 95% confidence intervals (CI) around the estimated effect were evaluated (Guyatt, Oxman, Kunz, Brozek, et al. 2011). A ‘not serious’ rating was assigned if the combined sample size was large (optimal size of at least 300) and the 95% CI for the effect estimate (odds ratio, OR) fell between 0.75 and 1.25, indicating no substantial benefit or harm. It was rated ‘serious’ if the combined sample size was less than 300 or if the 95% CI indicated significant benefit or harm (OR below 0.75 or above 1.25), and ‘very serious’ if the sample size was under 300 and the 95% CI suggested substantial benefit or harm.

‘Publication bias’ was assessed by visually inspecting funnel plots and using Egger's regression test to detect asymmetry, provided there were at least 10 studies in the meta‐analysis (Guyatt, Oxman, Montori, Vist, et al. 2011). If quantitative analysis of publication bias could not be performed, qualitative factors such as study publication in smaller or prestigious journals and private funding were also evaluated (Guyatt, Oxman, Montori, Vist, et al. 2011).

## 3 Results

### 3.1 Study Selection

**FIGURE 1:**
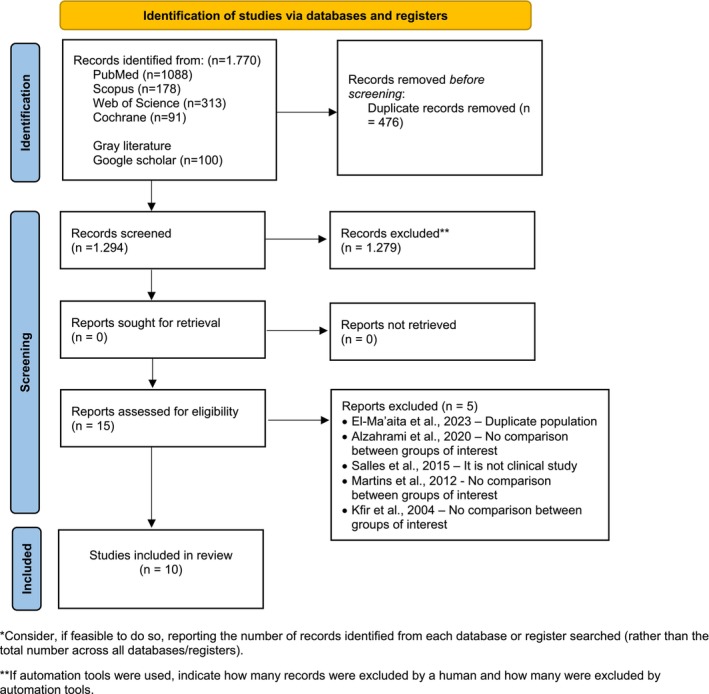
PRISMA diagram for study selection.

The initial search yielded 1770 studies (Table S1, Figure 1). After removing duplicates, 1294 studies were screened based on their titles and abstracts. Of these, 15 studies were identified as potentially eligible and were reviewed in full. Following the application of the eligibility criteria, five studies were excluded with reasons (Figure 1). Ultimately, 10 studies were included in this systematic review (Almanei 2018; Cheung and Liu 2009; El‐Ma'aita et al. 2024; Haug et al. 2018; Kelbauskas et al. 2009; Miçooğulları Kurt et al. 2022; Marinova et al. 2021; Matoug‐Elwerfelli et al. 2022; Tekín et al. 2023; Zajkowski et al. 2020).

### 3.2 Data Extraction

Table 1 and Table S2 summarise the main characteristics of the included studies, while Table 2 summarises the results of the included studies.

**TABLE 1:** Main results of the included studies.

Author and year	Manual file (control group): sample size	Mechanic file: sample size	Measured outcomes and results	Parameters used for outcome assessment	Main findings
Almanei (2018)	Kfile (Dentsply, Tulsa, OK, USA): 23 teeth	ProFile (DentsplyMaillefer, OK, USA): 23 teeth	Obturation length was significantly better in teeth prepared by ProFile rotary technique (65.5%) than by hand preparation (34.5%). Procedural errors were significantly more frequent in teeth prepared with hand files (68.2%) compared to ProFile rotary files (31.8%)	Obturation length: adequate (0–2 mm short of the apex) and inadequate (short > 2 mm or overfilled) Obturation density: adequate (0–2 voids) and inadequate (> 2 voids) Obturation taper: adequate (Consistent taper from coronal to apical third) and inadequate (Inconsistent taper from coronal to apical third) Procedural errors: dichotomic (yes or no) Overall quality: acceptable with no procedural errors and acceptable with procedural errors/not acceptable and require retreatment	The quality of root canal treatment was better in teeth prepared by ProFile rotary technique than by hand preparation
Cheung and Liu (2009)	Flexofile (Dentsply Maillefer): 115 M	ProFile: 110 M	The rotary instrumentation group was associated with a higher rate of complete periapical healing (77% vs. 60%) and a lower incidence of procedural errors (19% vs. 39%) when compared to the manual instrumentation group	Treatment outcome: favourable (No signs/symptoms and no periapical lesion or diminishing lesion (follow‐up < 4 years)), uncertain (Stable preexisting lesion without symptoms (follow‐up < 4 years)) or failure to heal (New/enlarging lesion or persistent radiolucency ≥ 4 years after treatment). Procedural errors (ledging, perforation (lateral or strip perforation), apical transportation, stripping (but not perforated), and fractured instrument): dichotomic favourable or uncertain	The use of rotary instruments resulted in a lower incidence of procedural errors and a better treatment outcome
El‐Ma'aita et al. (2024)	NR*: 109	ProTaper Gold (Dentsply Maillefer, Baillagues, Switzerland): 104	Rotatory instrumentation resulted in more sufficient treatment compared with manual instrumentation (49% vs. 30.3%)	Treatment outcome: sufficient or insufficient Procedural errors: under extension (> 2 mm short of the apex), over extension (beyond the radiographic apex), improper apical instrumentation size (too large or too small for that root), ledge, canal transportation, missed canal, access cavity perforation, instrumentation perforation, strip perforation, separated instrument, sealer extrusion, insufficient obturation (root canal space with resultant voids), improper access cavity (over or under‐drilled)	Rotary instrumentation improved the quality of root canal treatment compared to manual instrumentation
Haug et al. (2018)	K‐file (Dentsply Maillefer), K‐flexofile (Dentsply Maillefer), and K‐file NiTi flexfile (Dentsply Maillefer): 141	WaveOne (Dentsply Maillefer, Ballaigues, Switzerland): 116	No significant differences in endodontic mishapes were observed between the different instrumentation techniques. Mishaps were influenced by case difficulty. Five WaveOne files had instrument separations vs. one hand file	Endodontic mishaps: instrument separation, loss of working length, canal transportation, overinstrumentation (beyond apical foramen), short obturation, lateral or strip perforation, obturation more than 2 mm from the radiographic apex, and overfill with gutta‐percha (beyond the apical foramen)	There were no significant differences between endodontic mishaps and instrumentation techniques
Kelbauskas et al. (2009)	K‐file: 120	ProTaper: 138	The homogeneity of the filling material in root canals was statistically better in canals instrumented by ProTaper rotary (93,3%) compared to those by hand files (71,4%). There was no significant difference regarding overfilling between ProTaper group (5.1%) and hand file group (5.8%)	Obturation quality: Root canal filling to the radiographic apex—acceptable (within 2 mm of the radiographic apex), over‐filled (extruded beyond the radiographic apex) and under‐filled (> 2 mm short). Density of the filling material—good homogeneity (uniform throughout the lumen of the root canal), poor homogeneity (non‐uniform). Root fillings: adequate (without visible voids contained within the tooth and ending no less than 2 mm) and inadequate (underfilled, overfilled or poorly condensed)	The filling density and homogeneity were statistically better in canals prepared using ProTaper systemA
Miçooğulları Kurt et al. (2022)	K‐file (Mani): 245	WaveOne: 247	Procedural errors were more frequent in hand group (42%) compared to the reciprocating group (30.4%), without statistically significant difference. Inadequate filling was significantly more frequent in hand group compared to reciprocating file group (11.4% vs. 1.2%). Over‐instrumentation was more frequent in WaveOne group (9.3%) compared to hand group (1.2%)	Obturation quality: underfilling (> 2 mm short of the radiographic apex), overfilling, and inadequate root canal filling (not obturated root canal or presence of visible voids) Procedural errors: ledge, perforation, instrument fracture, overinstrumentation	Reciprocating instruments showed better technical quality in preparation and obturation, especially in difficult cases. However, over‐instrumentation was more common in WaveOne group
Marinova et al. (2021)	NR*: 652	Pro Taper Universal: 175	Excellent results in root canal filling were observed in 57% of hand instrumentation group and 76% in ProTaper group. More overfilled teeth were observed when rotary instrumentation was used (12.5%) compared with 9.4% for the hand instrumentation	Root canal filling: Length of the root canal filling: good (0–1 mm from the radiographic apex), acceptable (1–3 mm from the radiographic apex), unacceptable (than 3 mm from the radiographic apex) and overfilled (extending beyond the radiographic apex). Registered procedural errors: ledges, missed canals, apical transportation, perforations and fractured instruments	Cleaning and shaping of the root canals with rotary instruments were associated with significantly higher percentage of teeth with excellent length of the root canal filling. However, overfilling was most commonly observed when rotary instruments were used
Matoug‐Elwerfelli et al. (2022)	K‐file (Mani): 366	ProTaper Universal: 109	There was significant more frequent instrumentation mishapes in teeth instrumented by hand files (45.4%) compared to the rotary files (38.5%), as well as more frequent obturation failure (48.4% vs. 37.6%)	Instrumentation related mishaps: ledges, zipping, instrument separation, perforation (furcation, strip or root), Obturation related mishaps: length of root canal filling: acceptable (2 mm short of the radiographic apex) or unacceptable (< 2 mm short of radiographic apex). Density of root canal filling: acceptable (uniform density without voids and canal space is not visible) or unacceptable (poor density with the presence of voids and visible canal space)	Rotary instrumentation reduced instrumentation and obturation mishaps
Tekín et al. (2023)	NR*: 285	VDW rotary files: 280	The rotary group had a significantly higher success rate (75%) compared to the manual group (53.7%), as well as lower frequency of procedural errors (6.4% vs. 12.3%)	Treatment quality and procedural errors: ledge formation, apical transportation, apical perforation, furcation perforation, strip perforation, instrument fracture, zip formation, overfilling (extruded root filling from the apex), underfilling (2 mm or shorter than the radiologic apex) and voids (presence of the voids in obturation)	Rotary instrumentation demonstrated higher technical quality and fewer procedural errors compared to manual technique
Zajkowski et al. (2020)	NR*: 46	WaveOne Gold or Reciproc: 132	Absence of postoperative pain was observed in 78.3% of manual cases and 83.3% of reciprocating cases, without statistically significant difference	Postoperative pain: 0 = absent; 1 to 4 = mild pain; 5 to 7 = moderate pain; 8 to 10 = severe pain	No association was found between postoperative pain and the instrumentation technique used. Previous symptomatology, however, was linked to higher frequency and intensity of postoperative pain

Abbreviation: NR*: non‐reported.

**TABLE 2:** Main results of included studies (number of patients in the group/percentage).

Author (year)	Almanei (2018)	Cheung and Liu (2009)	El‐Ma'aita et al. (2024)	Haug et al. (2018)	Kelbauskas et al. (2009)	Marinova et al. (2021)	Matoug‐Elwerfelli et al. (2022)	Miçooğulları Kurt et al. (2022)	Zajkowski et al. (2020)	Tekín et al. (2023)
Endodontic Mechanical Instrumentation	Root canal filling length: Adequate: 19 (65.52%) Inadequate: 4 (23.53%) Root canal filling density: Adequate: 20 (51.28%) Inadequate: 3 (42.86%) Root canal filling taper: Adequate: 19 (47.50%) Inadequate: 4 (66.67%) Procedural errors: Yes: 7 (31.82%) No: 16 (66.67%) Overall quality: Acceptable with no procedural errors: 16 (66.67%) Acceptable with procedural errors/not acceptable and require retreatment: 7 (31.82%)	Treatment outcome: Favourable: 65 (73%) Uncertain: 9 (10.1%) Failure to heal: 15 (16.8%) Procedural errors: 19%	Treatment outcome: Sufficient: 49% Procedural errors: NA*	Instrument separation: 1.94% Procedural errors: NA*	Root canal filling length: Acceptable: 86.2% Over‐filled: 5.1% Under‐filled: 8.7% Root canal filling homogeneity and length: acceptable: 93.3% Good homogeneity and under‐filled: 75% Good homogeneity and over‐filled: 85.7%	Root canal filling length: Good: 133 (76%) Acceptable: 12 (6.9%) Unacceptable: 2 (1.1%) Overfilled: 28 (16%) Procedural errors: NA*	Ledge: 3 (2.8%) Zipping: 4 (3.7%) Perforation: 34 (31.2%) Instrument separation: 3 (2.8%) Overall instrumentation mishaps: 42 (38.5%) Overall obturation mishaps: 41 (37.6%)	Technically acceptable: 172 (69.6%) Underfilling: NA* Overfilling: 14.7% Inadequate root canal filling: 1.2% Overinstrumentation: 9.3% Ledge: NA* Instrument fracture: NA* Strip perforation: 1 (0.2%)	Postoperative pain: Absent: 110 (83.3%) Mild: 11 (8.3%) Moderate: 4 (3%) Severe: 7 (5.3%)	Successful: 210 (75%) Root canal filling quality: Adequate: 81.1% Acceptable: 6.4% Underfilling: 5.4% Overfilling: 7.1% Homogeneity of root canal filling: Non homogenous: 40 (14.3%) Homogeneous: 240 (85.7%) Outcome of root canal treatment: Unsuccessful: 25% Successful: 75% Procedural errors: 6.4% Broken instruments: 1.8% Apical perforation: 4 (1.4%) Apical transportation: 3.6% Ledge formation: 1.4%
Manual Endodontic Instrumentation	Root canal filling length: Adequate: 10 (34.48%) Inadequate: 13 (76.47%) Root canal filling density: Adequate: 19 (48.72%) Inadequate: 4 (57.14%) Root canal filling taper: Adequate: 21 (52.50%) Inadequate: 2 (33.33%) Procedural errors: Yes: 15 (68.18%) No: 8 (33.33%) Overall quality: Acceptable with no procedural errors: 8 (33.33%) Acceptable with procedural errors/not acceptable and require retreatment: 15 (68.18%)	Treatment outcome: Favourable: 36 (50.7%) Uncertain: 6 (8.4%) Failure to heal: 29 (40.8%) Procedural errors: 39%	Treatment outcome: Sufficient: 30.3% Procedural errors: NA*	Instrument separation: 0.39% Procedural errors: NA*	Root canal filling length: Acceptable: 81.7% Over‐filled: 5.8% Under‐filled: 12.5% Root canal filling homogeneity and length: Good homogeneity and acceptable: 71.4% Good homogeneity and under‐filled: 46.7% Good homogeneity and over‐filled: 57.1%	Root canal filling length: Good: 377 (57.8%) Acceptable: 147 (22.5%) Unacceptable: 54 (8.3%) Overfilled: 74 (11.3%) Procedural errors: NA*	Ledge: 17 (4.6%) Zipping: 13 (3.6%) Perforation: 135 (36.9%) Instrument separation: 10 (2.7%) Overall instrumentation mishaps: 166 (45.4%) Overall obturation mishaps: 177 (48.4%)	Technically acceptable: 142 (58%) Inadequate root canal filling: 11.4% Underfilling: NA* Overinstrumentation: 1.2% Ledge: NA* Instrument fracture: NA* Strip perforation: 0 (0%)	Postoperative pain: Absent: 36 (78.3%) Mild: 6 (13%) Moderate: 2 (4.3%) Severe: 2 (4.3%)	Successful: 153 (53.7%) Root canal filling quality: Adequate: 71.9% Acceptable: 11.9% Underfilling: 8.4% Overfilling: 7.7% Homogeneity of root canal filling: Non homogenous: 95 (33.3%) Homogeneous: 190 (66.7%) Outcome of root canal treatment—Unsuccessful: 46.3% Successful: 53.7%% Procedural errors: 12.3% Broken instruments: 3.2% Apical perforation: 1.1% Apical transportation: 17 (6%) Ledge formation: 5.6%

Abbreviation: NA*: crude data not available.

The sample sizes ranged from 23 to 652 teeth. Most of the studies used K‐files for stainless‐steel hand instrumentation. Regarding engine‐driven instrumentation, rotary systems were used in 7 studies, while 3 studies employed reciprocating systems. Among the rotary systems, ProTaper Universal was the most frequently used, appearing in 3 studies (Kelbauskas et al. 2009; Marinova et al. 2021; Matoug‐Elwerfelli et al. 2022), followed by ProFile in 2 studies (Almanei 2018; Cheung and Liu 2009), ProTaper Gold in 1 study (El‐Ma'aita et al. 2024), and VDW rotary files in 1 study (Tekín et al. 2023). For reciprocating systems, WaveOne was used in 2 studies (Haug et al. 2018; Miçooğulları Kurt et al. 2022), and 1 study evaluated both WaveOne Gold and Reciproc systems (Zajkowski et al. 2020). The types of teeth assessed varied across studies, including anterior teeth, premolars, and molars; however, most cases were of minimal to moderate complexity. For hand instrumentation, the step‐back technique was predominantly used, while engine‐driven instrumentation was performed according to the specifications recommended by each file manufacturer. Only three studies reported the pulp status prior to root canal treatment, all including cases of both pulpitis and pulp necrosis. All studies reporting on the irrigant solutions used during root canal treatments utilized sodium hypochlorite, with concentrations ranging from 0.5% to 2.5%. The most used obturation technique was cold lateral condensation, and AH Plus was the most commonly used root canal sealer. Radiographic evaluations were consistently performed using periapical radiographs in all included studies. Only two studies reported the follow‐up time, with a minimum of 1 year.

Overall, the included studies demonstrated superior outcomes for engine‐driven NiTi instrumentation compared to stainless‐steel hand techniques. All studies evaluating root canal filling quality reported significantly better filling quality with root canals prepared with engine‐driven NiTi instruments than with stainless‐steel hand files (Almanei 2018; El‐Ma'aita et al. 2024; Kelbauskas et al. 2009; Miçooğulları Kurt et al. 2022; Marinova et al. 2021; Matoug‐Elwerfelli et al. 2022; Tekín et al. 2023). Two studies observed a significantly higher occurrence of over‐instrumentation (Miçooğulları Kurt et al. 2022) and overfilling (Marinova et al. 2021) with engine‐driven NiTi instruments, while two others found no significant differences in overfilling between the two techniques (Kelbauskas et al. 2009; Tekín et al. 2023). Additionally, four studies indicated a significantly lower incidence of procedural errors with engine‐driven NiTi instruments compared to stainless‐steel hand files (Cheung and Liu 2009; Miçooğulları Kurt et al. 2022; Matoug‐Elwerfelli et al. 2022; Tekín et al. 2023). However, one study found no significant difference in procedural errors between the two instrumentation techniques (Haug et al. 2018).

Regarding treatment outcomes, one study reported a higher frequency of periapical healing following root canal treatment performed with engine‐driven NiTi instruments compared to stainless‐steel hand files (Cheung and Liu 2009). Postoperative pain was evaluated in one study, which found no association between pain and the use of stainless‐steel hand instrumentation or engine‐driven NiTi instrumentation (Zajkowski et al. 2020).

### 3.3 Quality Assessment

**FIGURE 2:**
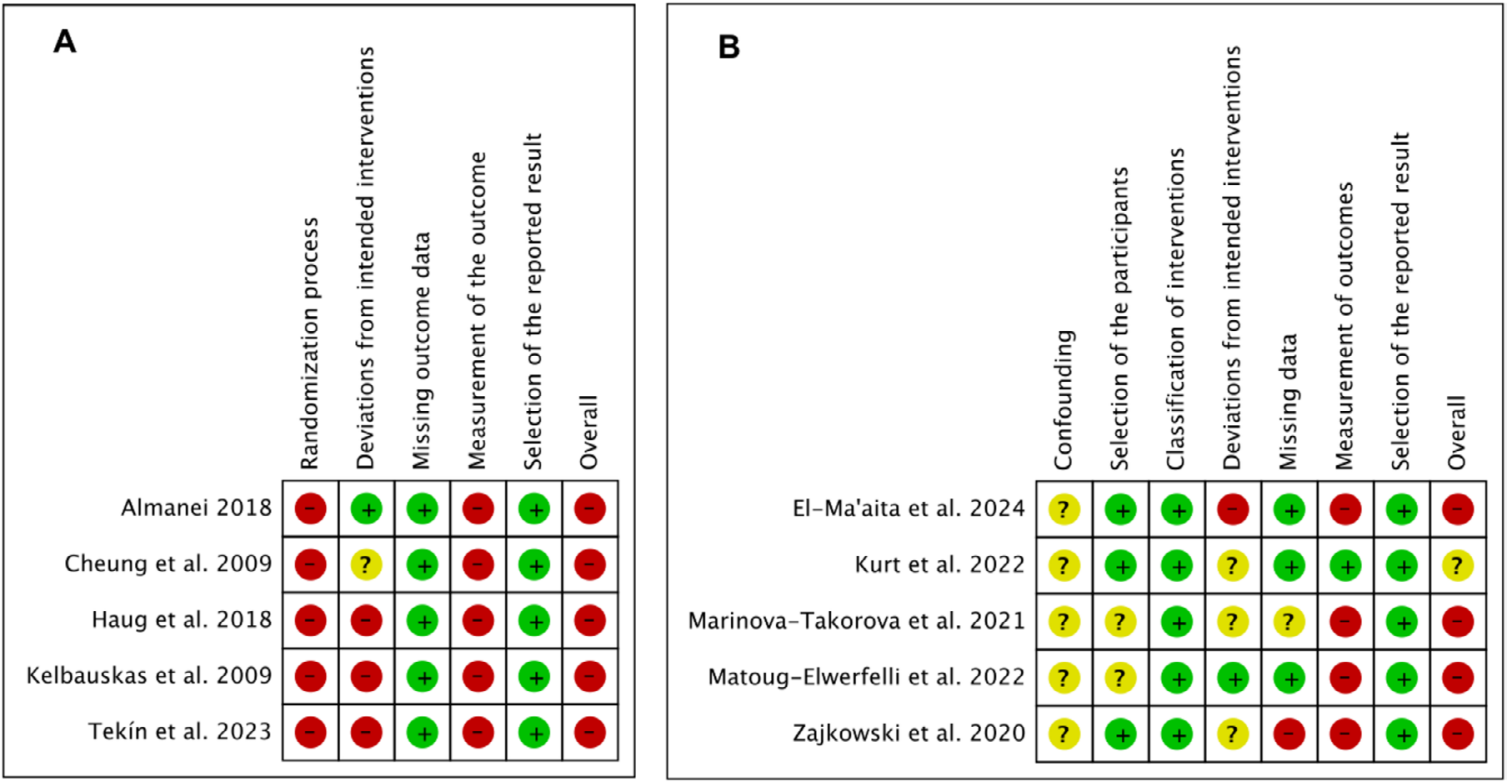
Risk of bias analyses for the included studies. (A) randomised clinical studies; (B) nonrandomized clinical studies.

The risk of bias analyses for the included studies are presented in Figure 2. Figure 2A illustrates the risk of bias for randomised clinical studies, while Figure 2B depicts the risk of bias for nonrandomized clinical studies. Among the included studies, nine were classified as having an overall high risk of bias (Almanei 2018; Cheung and Liu 2009; El‐Ma'aita et al. 2024; Haug et al. 2018; Kelbauskas et al. 2009; Marinova et al. 2021; Matoug‐Elwerfelli et al. 2022; Tekín et al. 2023; Zajkowski et al. 2020), and one study demonstrated a moderate risk of bias (Miçooğulları Kurt et al. 2022).

In randomized clinical trials (Almanei 2018; Cheung and Liu 2009; Haug et al. 2018; Kelbauskas et al. 2009; Tekín et al. 2023), biases were observed in the domains ‘Randomization process’ in all studies due to the absence of explanation on how randomization was performed; ‘Deviations from intended interventions’ due to lack of sample size calculation (Haug et al. 2018; Kelbauskas et al. 2009; Tekín et al. 2023) and lack of ethics committee consent (Cheung and Liu 2009); and ‘Measurement of the outcome’ in all studies due to the lack of blinded evaluation. In nonrandomized studies (El‐Ma'aita et al. 2024; Miçooğulları Kurt et al. 2022; Marinova et al. 2021; Matoug‐Elwerfelli et al. 2022; Zajkowski et al. 2020), biases were observed in the domains ‘Bias due to confounding’ in all studies due to the absence of patient's age evaluation, as age is an important prognostic factor for root canal treatment quality, affecting the root canal anatomy and the case difficulty; ‘Deviations from intended interventions’ due to lack of sample size calculation or absence of important data on sample size calculation (El‐Ma'aita et al. 2024; Miçooğulları Kurt et al. 2022; Marinova et al. 2021; Zajkowski et al. 2020); ‘Missing data’ due to the absence of information on describing the root canal treatment (Marinova et al. 2021), and due to lack of correspondence between initial sample size and treatments performed (Zajkowski et al. 2020); and ‘Measurement of the outcomes’ due to the lack of blinded evaluation (El‐Ma'aita et al. 2024; Marinova et al. 2021; Zajkowski et al. 2020).

### 3.4 Meta‐Analyses

**FIGURE 3:**
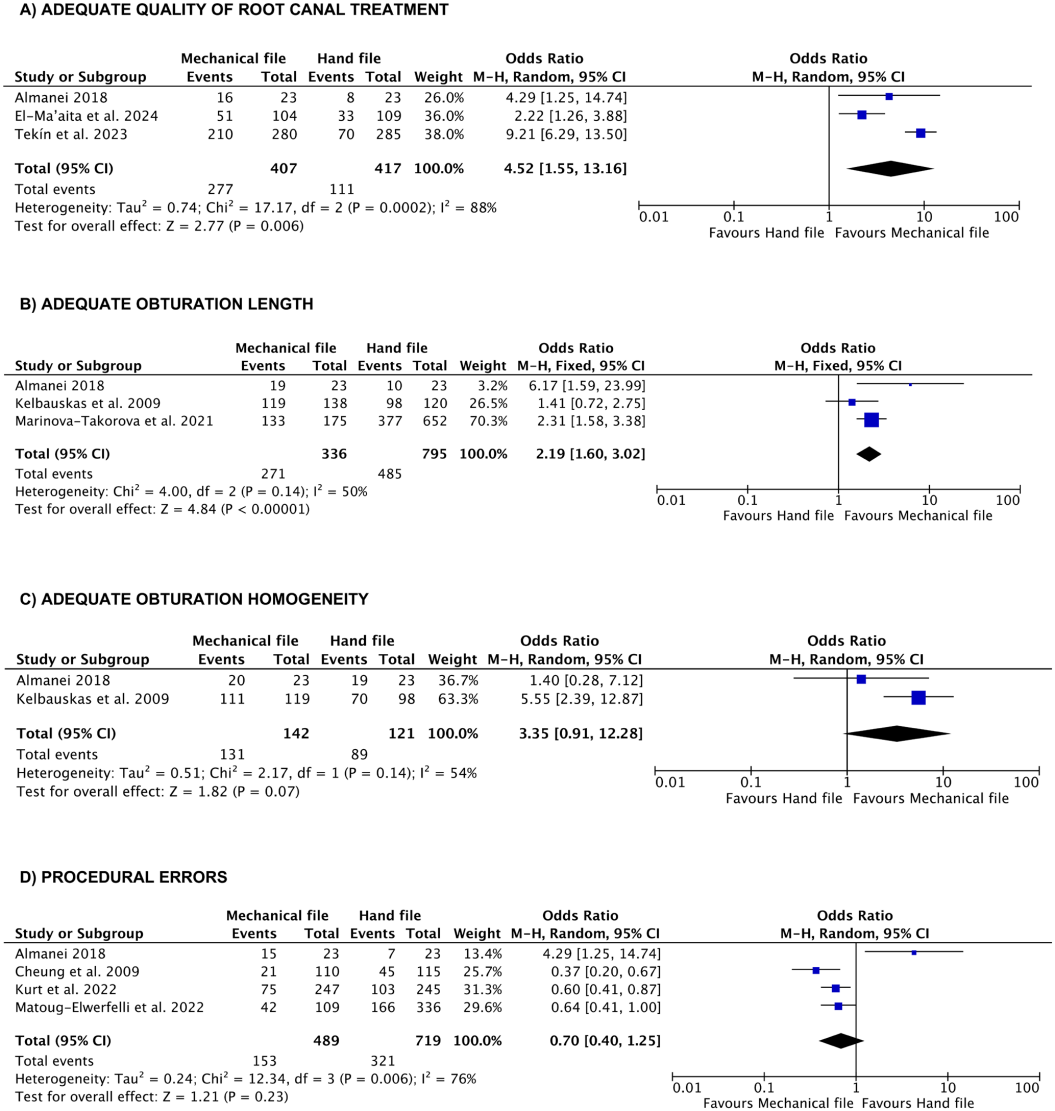
Forest plots for the meta‐analyses conducted to compare the prevalence of outcomes between engine‐driven and stainless‐steel hand instrumentation. (A) significantly better root canal filling length for engine‐driven root canal preparation compared to stainless‐steel hand instrumentation; (B) significantly higher root canal treatment quality for engine‐driven root canal preparation compared to stainless‐steel hand instrumentation; (C) no significant differences between engine‐driven and stainless‐steel hand instrumentation in terms of adequate root canal filling homogeneity; (D) no significant differences in procedural errors between engine‐driven and stainless‐steel hand instrumentation.

**FIGURE 4:**
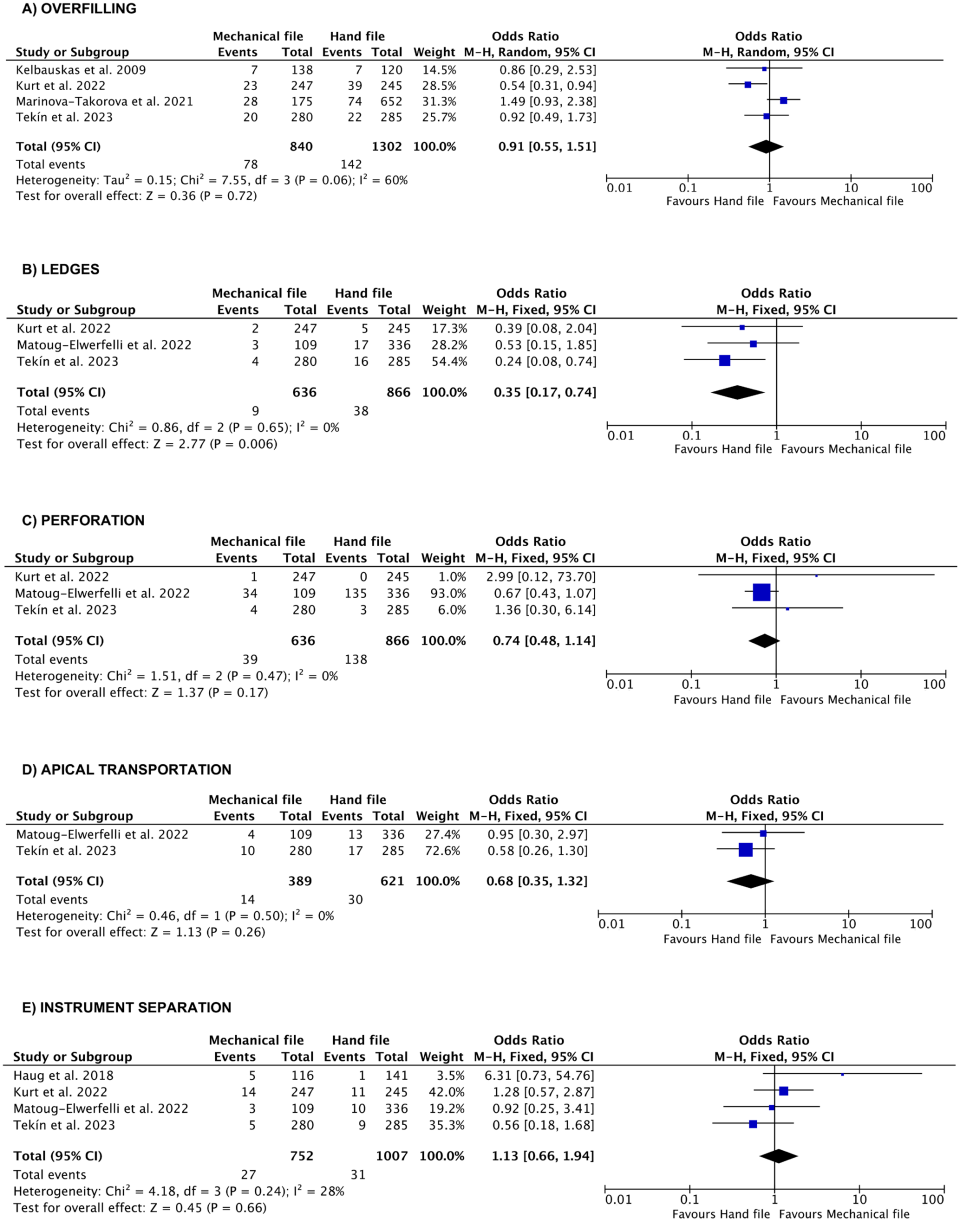
Additional meta‐analyses performed to evaluate the prevalence of specific procedural errors between the two instrumentation techniques. (A) no significant difference in the prevalence of overfilling between engine‐driven and stainless‐steel hand preparation; (B) significantly higher prevalence of ledges in stainless‐steel hand preparation compared to engine‐driven preparation, (C) no significant difference in the prevalence of perforation between engine‐driven and stainless‐steel hand preparation; (D) no significant difference in the prevalence of apical transportation between engine‐driven and stainless‐steel hand preparation; (E) no significant difference in the prevalence of instrument separation between engine‐driven and stainless‐steel hand preparation.

Meta‐analyses were conducted to compare the prevalence of several outcomes between engine‐driven and stainless‐steel hand instrumentation. Figure 3A illustrates the prevalence of adequate root canal treatment quality, while Figure 3B shows adequate root canal filling length; Figure 3C depicts adequate root canal filling homogeneity, and Figure 3D compares procedural errors. For procedural errors, additional meta‐analyses were performed to evaluate specific outcomes, including the prevalence of overfilling (Figure 4A), ledge formation (Figure 4B), perforation (Figure 4C), apical transportation (Figure 4D), and instrument separation (Figure 4E) between the two instrumentation techniques.

Figure 3A,B demonstrated significantly higher outcomes for engine‐driven root canal preparation compared to stainless‐steel hand instrumentation, with superior quality of root canal treatment (OR 4.52 [CI: 1.55, 13.16, *p* = 0.0002]) and adequate root canal filling length (OR 2.19 [CI: 1.60, 3.02, *p* < 0.00001]). A random‐effect model was applied to the analysis of root canal treatment quality due to high heterogeneity, whereas a fixed‐effect model was used for root canal filling length, reflecting low heterogeneity. Conversely, Figure 3C,D showed no significant differences between engine‐driven and stainless‐steel hand instrumentation in terms of adequate root canal filling homogeneity (OR 3.35 [CI: 0.91, 12.28, *p* = 0.07]) and procedural errors (OR 0.70 [CI: 0.40, 1.25, *p* = 0.23]). Random‐effect models were employed for both analyses due to high heterogeneity.

Among the types of procedural errors, Figure 4A showed no significant difference in the prevalence of overfilling between engine‐driven and stainless‐steel hand preparation (OR 0.91 [CI: 0.55, 1.51, *p* = 0.72]). A random‐effect model was used due to high heterogeneity. On the other hand, Figure 4B showed a significantly higher prevalence of ledges in stainless‐steel hand preparation compared to engine‐driven preparation (OR 0.35 [CI: 0.17, 0.74, *p* = 0.006]). Figure 4C–E showed no significant difference in the prevalence of perforations (OR 0.74 [CI: 0.48, 1.14, *p* = 0.17]), apical transportation (OR 0.68 [CI: 0.35, 1.32, *p* = 0.26]), and instrument separation (OR 1.13 [CI: 0.66, 1.94, *p* = 0.66]) between engine‐driven and stainless‐steel hand preparation. Fixed‐effect models were used since low heterogeneity was observed.

### 3.5 Grading of Evidence

The certainty of evidence for the included studies was overall rated as low quality (Table 3). The studies were classified as having a ‘very serious’ risk of bias, as most exhibited multiple issues in the risk of bias analyses (Guyatt, Oxman, Vist, Kunz, et al. 2011). However, they received a ‘not serious’ classification for the ‘Inconsistency’ domain, as the results across studies were consistent, with no unexplained heterogeneity observed (Guyatt, Oxman, Kunz, Woodcock, et al. 2011b).

**TABLE 3:** Assessment of certainty of evidence.

Certainty assessment						
Number of studies	Risk of bias	Inconsistency	Indirectness	Imprecision	Publication bias	Overall certainty of evidence
RCTs (*n* = 5) Non‐RCTs (*n* = 5)	Very serious^a^	Not serious^b^	Not serious^c^	Serious^d^	Not serious^e^	⨁⨁◯◯ LOW

^a^ Almost all studies showed high risk of bias.

^b^ All studies showed consistent results and did not observe unexplained heterogeneity.

^c^ Populations were representative of the patients for whom the interventions are recommended, and patient‐important outcomes were assessed.

^d^ In the meta‐analyses, the pooled sample size was lower than the optimal information size, and some 95% CI of the estimate of effect included appreciable benefit or harm.

^e^ Publication bias was not observed.

Similarly, the ‘Indirectness’ domain was rated as ‘not serious’ because the included populations were representative of patients for whom the interventions are recommended, and the assessed outcomes were relevant to patient care (Guyatt et al. 2011a). Conversely, the domain of ‘Imprecision’ was rated as ‘serious,’ as most meta‐analyses did not meet the required optimal information size, and some 95% confidence intervals (CIs) of the estimated effects (OR) included both potential harm and benefit (Guyatt, Oxman, Kunz, Brozek, et al. 2011).

‘Publication bias’ could not be quantitatively assessed due to the limited number of studies in the meta‐analyses, which made it impractical to evaluate funnel plots or perform regression‐based assessments (Guyatt, Oxman, Montori, Vist, et al. 2011). While publication bias cannot be entirely excluded, it was not considered sufficient to downgrade the quality of evidence, as the included studies originated from both prestigious and smaller journals, and none were funded by private sectors.

## 4 Discussion

In recent years, many universities have integrated engine‐driven NiTi preparation techniques into their undergraduate endodontic curricula (Alotaibi 2023; Baharin and Omar 2021; Nagendrababu et al. 2024; Segura‐Egea et al. 2021). These techniques are widely recognised for enhancing the efficiency and precision of root canal procedures (Buerklein and Arias 2023; Chaniotis and Ordinola‐Zapata 2022; Peralta‐Mamani et al. 2019). However, the transition from traditional stainless‐steel hand techniques to engine‐driven systems introduces a paradigm shift that demands not only technical adaptation but also a re‐evaluation of teaching methodologies and clinical protocols. While these innovations bring undeniable benefits, they also require careful implementation to avoid unintended consequences, such as increased procedural errors or compromised treatment outcomes.

The findings of this systematic review revealed that root canal preparation with rotary and reciprocating files resulted in significantly higher‐quality root canal treatment and better root canal filling quality, along with a lower incidence of ledge formation (Table 1, Figures 3 and 4). However, no significant differences were identified between the techniques in terms of overfilling, perforations, apical transportation, or instrument separation (Figure 4). Consequently, the null hypothesis—that there is no significant difference between engine‐driven and stainless‐steel hand root canal preparation in the quality of root canal treatments performed by undergraduate students—was rejected.

Intraoperative complications, such as instrument separation, loss of working length, or canal transportation, can significantly compromise the healing and prevention of apical periodontitis (Johnsen et al. 2023). These procedural errors may result in inadequate cleaning and filling, leaving the canal susceptible to reinfection. A systematic review and meta‐analysis focused on the quality of root canal treatment performed by undergraduate students using only stainless‐steel hand instrumentation (Ribeiro et al. 2018) highlighted that students relying on hand files are particularly prone to procedural errors and produce treatments of poorer technical quality. However, the occurrence of endodontic mishaps is often linked to the level of case difficulty, with procedures involving complex root canal anatomy, such as molars, posing significant challenges that demand greater precision and skill (Johnsen et al. 2023). The studies included in this review indicated that procedural errors were more influenced by case difficulty than by the choice of shaping technique (Haug et al. 2018; Miçooğulları Kurt et al. 2022). Notably, the use of engine‐driven instruments was associated with improved quality of root canal treatment, particularly in complex cases (Miçooğulları Kurt et al. 2022).

One crucial factor influencing the long‐term success of root canal treatment—specifically healing and the prevention of apical periodontitis—is the quality of root canal filling, particularly its length and density. All included studies evaluating filling quality reported significantly better filling outcomes with engine‐driven instruments compared to stainless‐steel hand techniques (Almanei 2018; El‐Ma'aita et al. 2024; Kelbauskas et al. 2009; Miçooğulları Kurt et al. 2022; Marinova et al. 2021; Matoug‐Elwerfelli et al. 2022; Tekín et al. 2023). Additionally, the meta‐analyses demonstrated that engine‐driven preparation achieved superior filling length compared to stainless‐steel hand instrumentation, although no statistically significant difference was observed in root canal filling density. It is important to note, however, that the observed improvements in filling outcomes may not be solely attributable to the preparation system but could also be influenced by the filling technique employed. While the engine‐driven instruments likely contribute to better shaping and cleaning of the root canal, which facilitates filling, the actual quality of the root canal filling is predominantly determined by the root canal filling method itself. Techniques such as warm vertical compaction or single‐cone obturation, which were not consistently detailed across studies, can significantly impact outcomes. Proper root canal filling plays a vital role in preventing microleakage and reinfection, reducing the risk of apical periodontitis, and supporting improved healing outcomes (Gulabivala and Ng 2023). These findings highlight the potential of engine‐driven NiTi instrumentation to enhance critical aspects of treatment quality, but they also underscore the need for future studies to standardise and report filling techniques to isolate the impact of preparation methods more accurately.

Training dental students in endodontics is a multifaceted process that demands the integration of technical skills and clinical experience. However, proficiency in endodontics extends beyond technical mastery; it requires a comprehensive understanding of case complexity, the influence of pre‐existing conditions on treatment outcomes, and the ability to make evidence‐based decisions. The recent guidelines from the European Society of Endodontology for undergraduate endodontic education (Baaij et al. 2024) highlight the importance of providing students with comprehensive clinical experience and emphasize training students to manage a variety of teeth—including anterior, premolar, and molar—across both pre‐clinical and clinical settings. Furthermore, the guidelines advocate for competency‐based education, prioritizing the development of skills that enable students to assess treatment complexity, evaluate tooth restorability, and determine when a case requires referral to a specialist (Baaij et al. 2024). By implementing a well‐structured curriculum that combines theoretical knowledge with hands‐on clinical practice, dental students can develop the skills and confidence needed to perform successful root canal treatments, ultimately contributing to long‐term periapical healing and teeth survival.

One of the primary limitations of this systematic review and meta‐analysis was the lack of standardisation in measured outcomes and several key variables across studies, which limited precise comparisons and reduced the number of studies eligible for each meta‐analysis. Subgroup analyses based on factors such as tooth type, case difficulty, the specific NiTi system, or the obturation technique could have provided more nuanced insights into the efficacy of engine‐driven NiTi instruments, but the necessary data to perform such analyses were not consistently reported. Additional factors such as pulp status prior to treatment and the underlying reason for root canal therapy—whether due to trauma, caries, or intentional treatment—may have also influenced the technical difficulty experienced by undergraduate students. Notably, most of the included studies were conducted in cases of minimal to moderate difficulty, which may not fully reflect the challenges encountered in clinical reality. While engine‐driven instrumentation can facilitate efficiency and standardisation in undergraduate training, the development of manual skills remains essential for managing complex cases, particularly those with challenging anatomy or a higher risk of complications. Therefore, the development of hand instrumentation skills remains essential in undergraduate education, as they provide a foundation for managing complex cases.

These sources of variability may have contributed to heterogeneity in the outcomes and should be considered when interpreting the findings of this review. Moreover, the review primarily includes studies from specific geographic regions, and the lack of detailed demographic data (e.g., patient age, pulpal diagnosis) may limit the applicability of the findings to other contexts. As well, most studies did not specify a minimum follow‐up time, despite the well‐established importance of adequate follow‐up for evaluating the periapical status after root canal treatment. The absence of these data hinders the assessment of the clinical relevance of the findings and limits the ability to account for potential confounding factors. Additionally, nine out of ten included studies presented a high risk of bias, particularly in domains such as randomization, blinding, and sample size calculation, and the certainty of evidence was low, primarily due to the high risk of bias and imprecision in the meta‐analyses. The predominance of studies with high risk of bias and the low level of evidence in the GRADE assessment may limit the strength of the evidence and should be considered with caution when interpreting the results. Inadequate randomization can introduce selection bias, potentially overestimating or underestimating treatment outcomes. Similarly, lack of blinding may lead to performance or detection bias, particularly in subjective outcome assessments such as postoperative pain or radiographic evaluations. Furthermore, the absence of appropriate sample size calculations increases the risk of underpowered studies, which may produce inconclusive or misleading results. Another important aspect is that the quality of the root canal treatments was assessed by radiographic imaging. While radiographic evaluation remains a standard and practical method for assessing the technical quality of root canal treatments, it does not fully capture the complexity of canal instrumentation and root canal filling. This limitation should be acknowledged as the technical outcomes are primarily based on radiographic criteria, which—like all assessment methods—may involve some degree of subjectivity and are inherently limited in fully representing the outcomes. Collectively, these biases can compromise the internal validity of individual studies and, consequently, reduce the overall strength of the conclusions drawn in this systematic review. These limitations in the primary studies underscore the need for more rigorous well‐designed clinical trials to provide stronger evidence.

Despite these limitations, the current review has notable strengths. It employed a comprehensive search strategy across four major electronic databases and grey literature, without imposing restrictions or filters. The selection process, data extraction, and risk of bias assessments were conducted independently by two authors, ensuring methodological rigor. Furthermore, robust tools were utilized, including RoB‐2 and ROBINS‐I for assessing the risk of bias in randomized and nonrandomized studies, respectively, and the GRADE framework to evaluate the certainty of evidence. Additionally, meta‐analyses were performed to compare critical clinical outcomes, including the prevalence of adequate quality of root canal treatment, root canal filling length, root canal filling homogeneity, procedural errors, overfilling, ledge formation, perforations, apical transportation, and instrument separation between engine‐driven and stainless‐steel hand instrumentation techniques. These methodological strengths enhance the reliability and applicability of the review's findings, providing valuable insights for improving endodontic education and practice.

## 5 Conclusions

The use of engine‐driven NiTi instruments by undergraduate students improves the quality of root canal treatments, improving root canal filling quality and reducing the incidence of ledge formation compared to stainless‐steel hand files, without increasing the risk of other iatrogenic complications such as overfilling, perforations, apical transportation, or instrument separation.

## Author Contributions

Each author contributed equally in conception, data analysis, writing and critical appraisal.

## Conflicts of Interest

The authors declare no conflicts of interest.

## Supporting information


**Data S1:** iej70056‐sup‐0001‐DataS1.docx.

**Table S1:** Search strategy and findings of each database.

**Table S2:** Characteristics of the includes studies.

## Data Availability

The data that support the findings of this study are available from the corresponding author upon reasonable request.
