# Supplementary material for: Influence of Engine‐Driven NiTi Files on the Effectiveness and Technical Quality of Endodontic Treatment Performed by Undergraduate Students: A Systematic Review and Meta‐Analysis
Source: Int Endod J. 2025 Oct 26;59(6):968–85. doi: 10.1111/iej.70056 (PMC13158543; doi:10.1111/iej.70056)
Supplement: Supplementary file 2 — Table S1: Search strategy and findings of each database. [file IEJ-59-968-s003.docx]

**Supplementary table 1.** Search strategy and findings of each database.

| **Database** | **Search strategy** | **Findings** |
| --- | --- | --- |
| **PubMed** | #1: ((students[Mesh]  OR students, dental[Mesh] OR education, dental[Mesh] OR dental education OR education, dental, graduate[Mesh] OR education, graduate[Mesh] OR graduate education OR undergraduate student OR dentistry student OR dentistry education OR endodontic education OR preclinic student OR pre-clinical student OR teaching OR preclinical dental education OR novice operators OR beginner operators OR pre-doctoral OR preclinical training OR dental training)) | 2345424 |
|  | #2: ((rotary OR reciprocating OR reciprocation OR mechanized instrumentation OR mechanized instruments OR engine-driven OR protaper OR reciproc OR wave one)) | 139364 |
|  | #3: ((root canal therapy[Mesh] OR root canal preparation[Mesh] OR root canal OR endodontics[Mesh] OR endodontology OR pulpectomy[Mesh] OR endodontically-treated OR canal preparation OR instrumentation OR root canal treatment OR shaping OR endodontic treatment OR endodontic therapy OR instrument separation OR instrument failure OR instrument fracture OR  file fracture OR procedural errors)) | 1,620,914 |
|  | **#1 AND #2 AND #3** | **1088** |
| **Cochrane Library** | #1: students OR "students, dental" OR "education, dental" OR "dental education" OR "education, dental, graduate" OR "education, graduate" OR "graduate education" OR "undergraduate student" OR "dentistry student" OR "dentistry education" OR "endodontic education" OR "preclinic student" OR "pre-clinical student" OR teaching OR "preclinical dental education" OR "novice operators" OR "beginner operators" OR pre-doctoral OR "preclinical training" OR "dental training"   in Title Abstract Keyword | 70958 |
|  | #2: rotary OR reciprocating OR reciprocation OR "mechanized instrumentation" OR "mechanized instruments" OR engine-driven OR protaper OR reciproc OR "wave one" in Title Abstract Keyword | 3460 |
|  | #3: "root canal therapy" OR "root canal preparation" OR "root canal" OR endodontics OR endodontology OR pulpectomy OR endodontically-treated OR "canal preparation" OR instrumentation OR "root canal treatment" OR shaping OR "endodontic treatment" OR "endodontic therapy" OR "instrument separation" OR "instrument failure" OR "instrument fracture" OR  "file fracture" OR "procedural errors" in Title Abstract Keyword | 74250 |
|  | **#1 AND #2 AND #3** | **91** |
| **Scopus** | #1: TITLE-ABS-KEY("students") OR TITLE-ABS-KEY("students, dental") OR TITLE-ABS-KEY("education, dental") OR TITLE-ABS-KEY("dental education") OR TITLE-ABS-KEY("education, dental, graduate") OR TITLE-ABS-KEY("education, graduate") OR TITLE-ABS-KEY("graduate education") OR TITLE-ABS-KEY("undergraduate student") OR TITLE-ABS-KEY("dentistry student") OR TITLE-ABS-KEY("dentistry education") OR TITLE-ABS-KEY("endodontic education") OR TITLE-ABS-KEY("preclinic student") OR TITLE-ABS-KEY("pre-clinical student") OR TITLE-ABS-KEY(teaching) OR TITLE-ABS-KEY("preclinical dental education" OR TITLE-ABS-KEY("novice operators") OR TITLE-ABS-KEY("beginner operators") OR TITLE-ABS-KEY(pre-doctoral) OR TITLE-ABS-KEY("preclinical training") OR TITLE-ABS-KEY("dental training") | 2327313 |
|  | #2: TITLE-ABS-KEY(rotary) OR TITLE-ABS-KEY(reciprocating) OR TITLE-ABS-KEY(reciprocation) OR TITLE-ABS-KEY("mechanized instrumentation") OR TITLE-ABS-KEY("mechanized instruments") OR TITLE-ABS-KEY(engine-driven) OR TITLE-ABS-KEY(protaper) OR TITLE-ABS-KEY(reciproc) OR TITLE-ABS-KEY("wave one") | 126988 |
|  | #3: TITLE-ABS-KEY("root canal therapy") OR TITLE-ABS-KEY("root canal preparation") OR TITLE-ABS-KEY("root canal") OR TITLE-ABS-KEY(endodontics) OR TITLE-ABS-KEY(endodontology) OR TITLE-ABS-KEY(pulpectomy) OR TITLE-ABS-KEY(endodontically-treated) OR TITLE-ABS-KEY("canal preparation") OR TITLE-ABS-KEY(instrumentation) OR TITLE-ABS- KEY("root canal treatment") OR TITLE-ABS-KEY(shaping) OR TITLE-ABS-KEY("endodontic treatment") OR TITLE-ABS-KEY("endodontic therapy") OR TITLE-ABS-KEY("instrument separation") OR TITLE-ABS-KEY("instrument failure") OR TITLE-ABS-KEY("instrument fracture") OR  TITLE-ABS-KEY("file fracture") OR TITLE-ABS-KEY("procedural errors") | 12978 |
|  | **#1 AND #2 AND #3** | **178** |
| **Web of Science** | #1: TS=(students OR "students, dental" OR "education, dental" OR "dental education" OR "education, dental, graduate" OR "education, graduate" OR "graduate education" OR "undergraduate student" OR "dentistry student" OR "dentistry education" OR "endodontic education" OR "preclinic student" OR "pre-clinical student" OR teaching OR "preclinical dental education" OR "novice operators" OR "beginner operators" OR pre-doctoral OR "preclinical training" OR "dental training" ) | 1440791 |
|  | #2: TS=(rotary OR reciprocating OR reciprocation OR "mechanized instrumentation" OR "mechanized instruments" OR engine-driven OR protaper OR reciproc OR "wave one") | 76369 |
|  | #3: TS=("root canal therapy" OR "root canal preparation" OR "root canal" OR endodontics OR endodontology OR pulpectomy OR endodontically-treated OR "canal preparation" OR instrumentation OR "root canal treatment" OR shaping OR "endodontic treatment" OR "endodontic therapy" OR "instrument separation" OR "instrument failure" OR "instrument fracture" OR  "file fracture" OR "procedural errors") | 2025901 |
|  | **#1 AND #2 AND #3** | **313** |
