# Supplementary material for: Influence of Engine‐Driven NiTi Files on the Effectiveness and Technical Quality of Endodontic Treatment Performed by Undergraduate Students: A Systematic Review and Meta‐Analysis
Source: Int Endod J. 2025 Oct 26;59(6):968–85. doi: 10.1111/iej.70056 (PMC13158543; doi:10.1111/iej.70056)
Supplement: Supplementary file 3 — Table S2: Characteristics of the includes studies. [file IEJ-59-968-s002.docx]

**Supplementary table 2. Characteristics of the includes studies**

| **Author and year** | **Patients age** | **Tooth type** | **Case complexity** | **Pulpal diagnosis** | **Hand instrumentation technique** | **Irrigant solution** | **Obturation technique** | **Root canal sealer** | **Restorative material** | **Follow-up time** |
| --- | --- | --- | --- | --- | --- | --- | --- | --- | --- | --- |
| Almanei2018 | NR* | Maxillary and mandibular first and second molars | Minimum to moderate difficulty | NR* | Step‑back | 1% sodium hypochlorite | Cold lateral condensation | AH26 sealer | NR* | NR* |
| Cheung et al. 2009 | Average age: Rotary instruments: 43.3 years. Manual instruments: 40.5 years. | Maxillary and mandibular first and second molars | NR* | NR* | Step-back and step-down | 1%-2.5% hypochlorite solution | Lateral or vertical compaction | AH26 or AH Plus (Dentsply) | Amalgam and others | 21.9 ±  14.5 months |
| El-Ma’aita et al. 2024 | NR* | Molars | Low difficulty | NR* | Step-back | 1%-2.5% hypochlorite solution | Cold lateral condensation | AH Plus (Dentsply) | NR* | NR* |
| Haug et al. 2018 | NR* | Anteriors, premolars and molars | Minimal, moderate, or high | NR* | NR* | Dakin’s solution (0.5% buffered sodium hypochlorite solution). | Cold lateral condensation | AH Plus Sealer (Dentsply). | Intermediate restorative ﬁlling material (IRM Caps; Dentsply) | 1 year |
| Kelbauskas et al. 2009 | NR* | Single-rooted teeth and multi-rooted teeth | NR* | Pulpitis and pulp necrosis. | NR* | NR* | NR* | NR* | NR* | NR* |
| Kurt et al. 2022 | NR* | Anterior, premolar and molar | Minimal and moderate difficulty | Irreversible pulpitis and pulp necrosis. | Step-back | 2.5% sodium  hypochlorite (Merck, Darmstadt, Germany) | Cold lateral condensation | AD Seal (Meta Biomed, Cheongju, South Corea). | Resin composite (3 M ESPE, Dental Products, St. Paul, MN, USA). | NR* |
| Marinova-Takorova et al. 2021 | >18 years | Single-rooted teeth and multi-rooted teeth | NR* | Pulpitis and pulp necrosis | Step-back | NR* | NR* | NR* | NR* | NR* |
| Matoug-Elwerfelli et al. 2022 | NR* | Anterior and posterior teeth | Minimal and moderate difficulty | NR* | Step-back | 2.25% sodium hypochlorite. | Lateral condensation | AH Plus | NR* | NR* |
| Tekín et al. 2023 | NR* | Anterior, premolar and molar | NR* | NR* | Step-back | 2.5% sodium hypochlorite | Lateral condensation | AH Plus | NR* | NR* |
| Zajkowski et al. 2020 | NR* | Anterior, premolar and molar | NR* | NR* | Crown-down | 2.5% sodium hypochlorite | Lateral condensation | NR* | NR* | NR* |

NR*: non-reported.
